# Carrying SNP rs17506395 (T > G) in *TP63* gene and CCR5Δ32 mutation associated with the occurrence of breast cancer in Burkina Faso

**DOI:** 10.1515/biol-2022-0847

**Published:** 2024-04-03

**Authors:** Lassina Traoré, Mousso Savadogo, Abdou Azaque Zouré, Touwendpoulimdé Isabelle Kiendrebeogo, Fabienne Marie B. T. B. Soudre, Soayebo Dabre, Aida Djé Djénéba Traore, Marc Donald Wilfried Adico, Tilate Lare, Teega-Wendé Clarisse Ouedraogo, Rogomenoma Alice Ouedraogo, Abdoul Karim Ouattara, Edwige T. Yelemkoure, Alexis Yobi Sawadogo, Nayi Zongo, Hierrhum Aboubacar Bambara, Christelle W. Nadembega, Florencia W. Djigma, Jacques Simpore

**Affiliations:** Laboratory of Molecular and Genetic Biology (LABIOGENE), Joseph KI-ZERBO University, 03 BP 7021, Ouagadougou 03, Burkina Faso; Pietro Annigoni Biomolecular Research Centre (CERBA), 01 BP 364, Ouagadougou 01, Burkina Faso; Biomedical Research Laboratory (LaReBio), Biomedical and Public Health Department, Health Sciences Research Institute (IRSS/CNRST), 03 BP 7192 Ouaga 03, Burkina Faso; Laboratory Department, University Hospital Centre-Yalgado OUEDRAOGO, Joseph KI-ZERBO University, UFR/SDS, 03 BP 7021, Ouagadougou 03, Ouagadougou, Burkina Faso; Gaoua University Centre, NAZI BONI University, 01 BP 1091, Bobo-Dioulasso 01, Burkina Faso; Manga University Centre, Norbert ZONGO University, Koudougou, Burkina Faso; Gynecology Department, Yalgado Ouédraogo University Hospital, UFR/SDS, 03 BP 7021, Ouagadougou 03, Ouagadougou, Burkina Faso; Department of Surgery, Visceral and Digestive Surgery Service, Yalgado Ouédraogo University Hospital, Joseph KI-ZERBO University, UFR/SDS 03 BP 7021, Ouagadougou 03, Ouagadougou, Burkina Faso; Oncology Department, University Hospital Centre-BOGODOGO, Joseph KI-ZERBO University, UFR/SDS, 03 BP 7021, Ouagadougou 03, Ouagadougou, Burkina Faso

**Keywords:** breast cancer, *TP63*, CCR5, polymorphism, Burkina Faso

## Abstract

Genetic alterations in the *TP63* (GenBank: NC_000003.12, ID: 8626) and *CCR5* (receptor 5 chemokine co-receptor) (GenBank: NC_000003.12, ID: 1234) genes may increase the risk of developing breast cancer. The aim of this study was to investigate the probable involvement of polymorphisms rs17506395 in the *TP63* (tumour protein 63) gene and the CCR5Δ32 mutation in the occurrence of breast cancer in Burkina Faso. This case–control study included 72 patients and 72 controls. Genotyping of SNP rs17506395 (TP63) was performed by polymerase chain reaction–restriction fragment length polymorphism, and genotyping of the CCR5Δ32 mutation was performed by allele-specific oligonucleotide polymerase chain reaction. For SNP rs17506395 (TP63), the genotypic frequencies of wild-type homozygotes (TT) and heterozygotes (TG) were, respectively, 27.72 and 72.22% in cases and 36.11 and 63.89% in controls. No mutated homozygotes (GG) were observed. For the CCR5Δ32 mutation, the genotypic frequencies of wild-type homozygotes (WT/WT) and heterozygotes (WT/Δ32) were 87.5 and 13.5%, respectively, in the cases and 89.29 and 10.71%, respectively, in the controls. No mutated homozygotes (Δ32/Δ32) were observed. None of the polymorphisms rs17506395 of the *TP63* gene (OR = 1.47, 95% CI = 0.69–3.17, *P* = 0.284) and the CCR5Δ32 mutation (OR = 1.32, 95% CI = 0.46–3.77; *P* = 0.79) were associated with the occurrence of breast cancer in this study.

## 1 Introduction

Breast cancer is a major public health problem. In 2020, according to GLOBOCAN, it was the most frequently diagnosed cancer in women, causing the greatest loss of life in both developed and developing countries. In 2020, there were around 2,261,419 new cases and 684,996 deaths worldwide [1]. In Burkina Faso, over the same period, 1,927 new cases were diagnosed, compared with 1,142 deaths, making it the leading cause of death from cancer [1]. In addition, a number of studies have shown the involvement of socio-demographic, clinical, and behavioural factors (age, place of residence, occupation, and lifestyle) in the development of breast cancer [2]. Familial forms are hereditary and result from a genetic predisposition. However, sporadic forms are the result of an association between both genetic and environmental factors [2,3]. Polymorphisms or mutations in certain genes such as *BRCA1* and *BRCA2* [4,5] and *ATM*, *BRIP1*, *CHEK2*, *NBS1*, *PALB2,* and *RAD50* [6] are also incriminated in the development of breast cancer. In addition to these polymorphisms and mutations, the SNP rs17506395 (*TP63* gene) and the CCR5Δ32 mutation are thought to be involved in the development of breast cancer [7,8].

The *TP63* gene (OMIM:603273, HUGO HGNC:15979) belongs to the p53 family and is located on chromosome 3q28. It codes for the p63 protein, which plays a crucial role in the maintenance of stem cells in several epithelial tissues and is required for the normal development of epithelial organs, including the mammary glands [9]. The SNP rs17506395 (T > G) (189803530T > G,189521319T > G,211785T > G) of the *TP63* gene, which was initially associated with fertility, has also been implicated in the development of several cancers, including breast cancer [10,11,12].

The *CCR5* gene (OMIM:601373, HUGO HGNC:1606) is located on chromosome 3 and comprises three exons encoding the CCR5 protein. CCR5 is a chemokine receptor of the β-chemokine receptor family of integral membrane proteins [7]. A 32-base pair deletion of the *CCR5* gene (CCR5Δ32) leads to the formation of a non-functional receptor that causes significant defects in ligand-mediated chemotaxis and has been implicated in a variety of immune-mediated diseases [13,14]. CCR5 may have an indirect effect on cancer progression by controlling the antitumour immune response [7].

The CCR5delta32 mutation (GenBank: NM_001394783.1) has been studied in several cancers including skin cancer, bladder cancer and cancer of the liver [15], cervical cancer [16], osteosarcoma [17], breast cancer [18,19], and oral cancer [20]. These broad studies on different kinds of cancer, however, often suggest contradictory results that bring up the importance of further studying these mutations in different populations.

Genetic predisposition to breast cancer in the African population is less well studied [8]. In Burkina Faso, previous studies have focused on certain genetic factors and breast and prostate cancers, in particular HLA-DRB1*11 1*12, *TP53* and *CHEK2, BRCA1,* and 943ins10 [21,22,23]; exons of *BRCA1* and *BRCA2* [24]; *R462Q* (rs 486907) and *D541E* (rs 627928) of *RNASEL* gene [25]; Ser217Leu and Ala541Thr of *ELAC2* gene [26]; and ERCC1 (rs3212986) and ERCC2 (rs1799793, rs13181) [27]. However, no study has yet looked at the involvement or otherwise of the *TP63* and *CCR5* genes in the occurrence of cancer in Burkina Faso. However, a study has been conducted in Cameroon to elucidate the involvement of SNP rs17506395 in the development of breast cancer [8], and it was found that the rs17506395 of the *TP63* gene was not involved in the development of breast cancer (OR = 0.86, *P* = 0.1269). It is in this context that this study aimed to investigate the probable involvement of the SNP rs17506395 of the *TP63* gene and the CCR5Δ32 mutation in the occurrence of breast cancer in Burkina Faso.

This could contribute to not only knowledge of the genetic risk factors for breast cancer but also provide knowledge that could be used in a strategy to prevent the disease.

## 2 Materials and methods

### 2.1 Setting and study population

This was a descriptive and analytical case–control study in Ouagadougou, Burkina-Faso. The study population consisted of 144 women, 72 of whom were patients with breast cancer (cases) and 72 healthy women without breast cancer (controls) who attended consultations at two University Hospitals: Yalgado OUEDRAOGO (CHU-YO) and Bogodogo (CHU-B) and two medical centres with a surgical unit: Schiphra and Paul VI. Various biomolecular tests were carried out at the Laboratory of Molecular and Genetic Biology (LABIOGENE), Joseph KI-ZERBO University, and Pietro Annigoni Biomolecular Research Centre (CERBA). In this study, any participant who received anatomopathological confirmation was considered a “case.” Eligible controls were women with no breast abnormality on ultrasound examination. These controls shared the same socio-demographic framework as the patients in this study.

Any participant without anatomopathological confirmation of breast cancer and those with breast cancer who had not given their consent to take part in the study were excluded.


**Informed consent:** Informed consent has been obtained from all individuals included in this study.
**Ethical approval:** The research related to human use has been complied with all the relevant national regulations, institutional policies and in accordance with the tenets of the Helsinki Declaration, and has been approved by the Health Research Ethics Committee (CERS) of Burkina Faso (Deliberation No. 2019-5-067 of May 15, 2019).

### 2.2 Data collection and sampling

A questionnaire was administered to the patients to collect their socio-demographic, anthropometric, and clinical data. Five millilitres (5 mL) of venous blood was then collected in an EDTA (ethylene-diamine-tetra-acetic acid) tube and centrifuged at 3,500 rpm for 15 min. The plasma and pellet were stored separately at −20°C.

### 2.3 DNA extraction, quantification, and purity testing

The participant’s genomic DNAs were extracted using the QIAamp®DSP DNA Blood Mini kit (QIAGEN, GmbH, QIAGEN Strabe1, D-40724 Hilden, Germany). The Nanodrop (Thermo Fisher Scientific) was used to quantify and check the purity of the extracted DNA, the concentration of which was adjusted to 10 ng/µL.

### 2.4 Genotyping of SNP rs17506395 (T > G) in *TP63* and CCR5Δ32 mutation

For the rs17506395 polymorphism of the *TP63* gene, polymerase chain reaction-restriction fragment length polymorphism was used, and for the Δ32 mutation of the *CCR5* gene, allele-specific oligonucleotide polymerase chain reaction (ASO-PCR).

#### 2.4.1 SNP rs17506395 (T > G) in *TP63*

A reaction mixture with a total volume of 25 µL (per sample) composed of 4 µL of 5X FIREPOL^®^Master Mix (Solis BioDyne), 0.5 µL of each primer (0.5 µM), 14 µL of sterile water, and 5 µL of DNA (10 ng/µL). The sequences of the primer pairs used are recorded in Table 1 (Guleria et al., 2012; Tiofack et al., 2020). Amplification was carried out using the Gene Amp^®^PCR System 9700 thermal cycler (Applied Biosystems) following the amplification programme: initial denaturation at 94°C for 5 min followed by 40 amplification cycles (denaturation at 94°C, 30 s, hybridisation at 57°C for 30 s, and elongation at 72°C, 30 s), and final elongation at 72°C for 7 min. The PCR products were then enzymatically digested with the MboII enzyme at 37°C for 3 h. Finally, the digestion products were subjected to electrophoretic migration for 45 min (100 V) on a 2% agarose gel containing 2 µL of ethidium bromide (10 mg/mL) and visualised using the “Vilber” apparatus (vilber Lourmat).

**Table 1:** Primers for amplification of SNP rs17506395 (*T* > *G*) in *TP63* and CCR5Δ32 mutation

Polymorphism	Primers	Amplicons size (bp)
*TP63* (rs17506395)	F: 5′-ACA GAT AAA TTG GTG GAG AGA GAT-3′	450pb; After digestion: 215pb, 235pb and 450pb
	R: 5′-CAC TGT TTG GAC CCT GGAA- 3'	
*CCR5 (mutation* Δ32)	F: 5′-GTG GTG ACA AGT GTG ATC AC-3′	320pb and 288pb
	R: 5′-TTG TAG GGA GCC CAG AAG AG-3′	

#### 2.4.2 ASO-PCR genotyping of CCR5Δ32 mutation

Genotyping of the CCR5 polymorphism was carried out using the ASO-PCR technique. For the Δ32 mutation, genotyping of each sample was carried out in a 25 μL reaction medium containing 15 µL of pure water (molecular biology grade water), 04 µL 5X FIREPOL^®^Master Mix (Solis BioDyne) 5x, 0.5 µL of each primer (0.5 µM), and 5 µL of DNA (10 ng/µL). Table 1 shows the primer pairs used (Guleria et al., 2012). The thermocycling parameters were an initial denaturation at 94°C for 05 min followed by 50 amplification cycles, then 40 amplification cycles (denaturation at 94°C, 30 s, hybridisation at 55°C for 30 s, and elongation at 72°C, 30 s), and final elongation at 72°C, 7 min using the Gene Amp^®^PCR System 9700 (Applied Biosystems).

The PCR products were then electrophoresed on a 2% agarose gel, migrated for 45 min (100 volts), and visualised using the “Vilber” apparatus (Vilber Lourmat).

### 2.5 Statistical analysis

Data were entered using Excel 2016 and analysed using SPSS software version 21.0, R software version 4.2.1, and Epi Info software version 7. The chi-square test was used for frequency comparisons. Odds ratios (OR) and 95% confidence intervals (CI) were calculated to assess risk. Results were considered statistically significant for a *P*-value of less than 0.05.

## 3 Results

### 3.1 Clinical and socio-demographic characteristics

Our study included 144 women: 72 patients and 72 controls. Age ranged from 19 to 70 years, with an average of 41.08 ± 12.19 years. The majority of patients (68.06%) were aged strictly over 40. The majority of participants lived in urban areas (93.06% for cases and 100% for controls).

Body mass index (BMI) was calculated according to the US National Institute of Health/National Heart Lung and Blood Institute criteria. A statistically significant association was found between obesity and the occurrence of breast cancer (OR = 3.88, 95% CI = 1.13–14.38, *P-*value = 0.015). There was no significant association between late menopause and the occurrence of breast cancer (Table 2). Approximately 15.27% of patients had a family history of breast cancer.

**Table 2:** Clinical characteristics of the study population

	Cases	Controls	OR (95% CI)	
	*N* = 72 (%)	*N* = 72 (%)		*P*-value
**BMI (kg/m** ^**2**^)				
Lean/normal	6 (8.33)	22 (30.56)	—	Reference
Overweight	10 (13.9)	17 (23.61)	2.15 (0.57–8.70)	0.203
Obesity	18 (25)	17 (23.61)	3.88 (1.13–14.38)	0.015
Other	38 (52.70)	16 (22.22)		
**Late menopause (age 55)**				
Yes	2 (2.78)	0 (00)		NA
No	70 (97.22)	72 (100)	—	Reference
**Family history**				
Yes	12 (17.10)	23 (31.94)	0.42 (0.19–0.94)	0.05
No	60 (82.9)	49 (68.06)	—	Reference

OR: odd ratio; 95% CI: 95% confidence interval; BMI: body mass index.

### 3.2 rs17506395 (*TP63*) polymorphism and risk of breast cancer

#### 3.2.1 Amplification and digestion results

**Figure 1:**
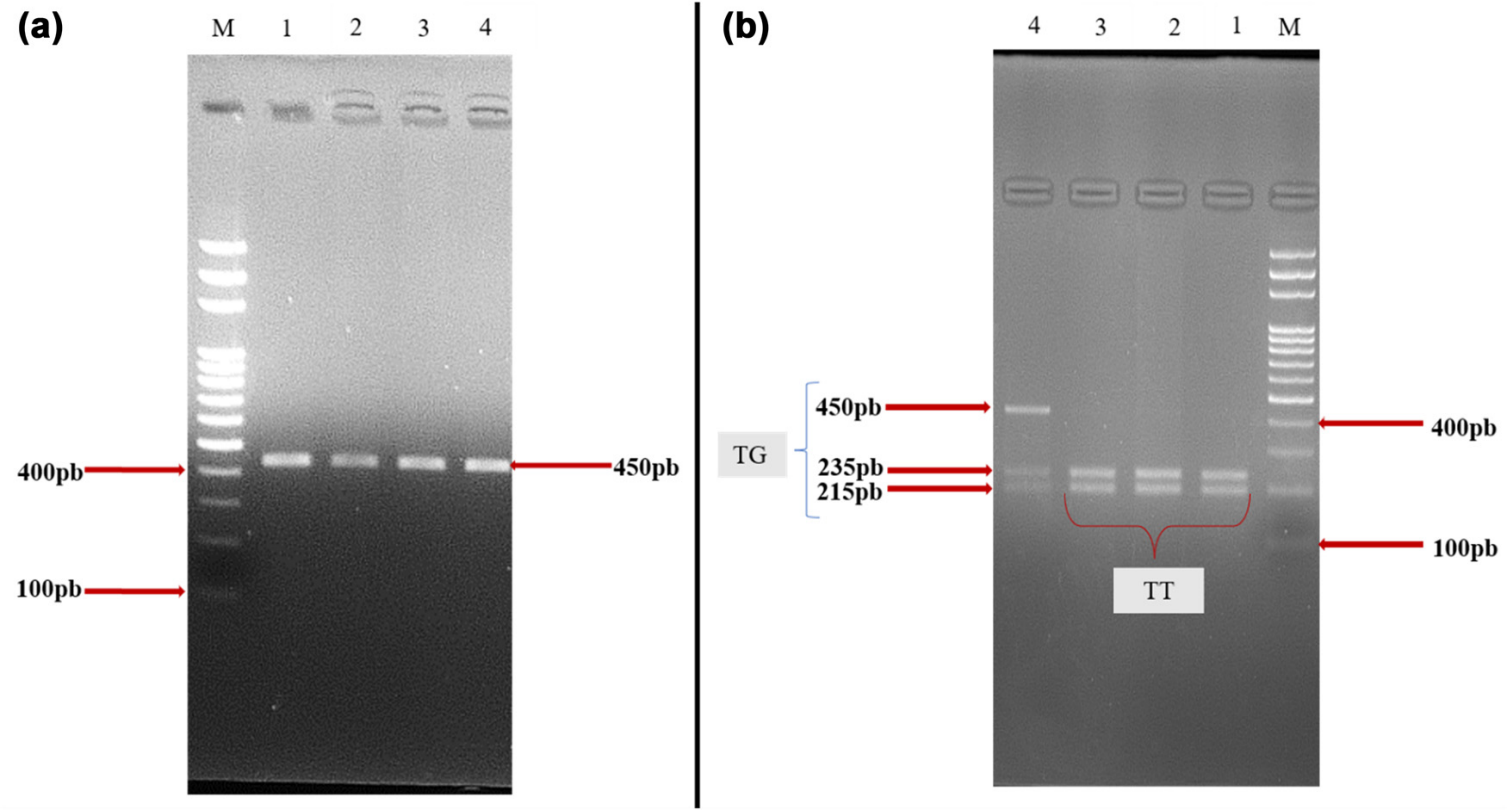
Electrophoretic profiles of the TP63 gene: Before (a) and after digestion (b). (M) 100bp molecular weight marker; (a) amplification result after conventional PCR; (b) amplification result after enzymatic digestion.

Conventional PCR of a fragment of the *TP63* gene yielded an amplicon at 450 bp (Figure 1a) and, after digestion, differentiated between homozygous wild-type individuals (TT): 235 and 215 bp; heterozygous individuals (TG): 215, 235, and 450 bp; and homozygous mutants (GG): 450 bp (Figure 1b). The results revealed either homozygous wild-type individuals (TT) or heterozygotes (TG). No mutant homozygotes were found in the study population (Figure 1b).

#### 3.2.2 Genotypic and allelic frequencies

The genotypic frequencies of rs17506395 (*TP63*) did not conform to the Hardy–Weinberg equilibrium (HWE) between patients (χ² = 23.27, *P*-value = 0.000001) and controls (χ² = 16.74, *P-*value = 0.000043). The frequency of the mutated allele [G] was 36.1% in cases and 32% in controls (Table 3). No statistically significant association was observed between this allele and the occurrence of breast cancer (OR = 1.20, 95% CI = 0.72–2.02, *P*-value = 0.456).

**Table 3:** Distribution of allelic and genotypic frequencies of TP63 and CCR5

	Cases	Controls	OR (95% CI)	*P*-value
	*N* = 72 (%)	*N* = 72 (%)		
***TP63*** **(rs17506395)**				
**Alleles**				
T	92(0.639)	98 (0.680)	—	Reference
G	52 (0.361)	46 (0.320)	1.20 (0.72–2.02)	0.456
**Genotypes**				
TT	20 (27.78)	26 (36.11)	—	Reference
TG	52 (72.22)	46 (63.89)	1.47 (0.69–3.17)	0.284
GG	0 (0.00)	0 (0.0)	NA	NA
***CCR5***				
**Alleles**				
WT	135(0.9375)	137 (0.951)	—	Reference
Δ32	9 (0.0625)	7 (0.049)	1.33 (0.41–4.46)	0.596
**Genotypes**				
WT/WT	63 (87.5)	65 (89.29)	—	Reference
WT/Δ32	9 (13.5)	7 (10.71)	1.32 (0.46–3.77)	0.79
Δ32/Δ32	0 (0.00)	0 (0.00)	NA	NA

OR: odds ratio; 95% CI: 95% confidence interval; N: number; *P*: *P*-value, NA: not applicable.

The results showed that the genotypic frequencies of wild-type homozygotes (TT) and heterozygotes (TG) were, respectively, 27.78% and 72.22% in patients and 36.11% and 63.89% in controls. No homozygous mutations (GG) were observed. No significant association was found between the variants of this polymorphism and breast cancer (G: OR = 1.20, CI = 0.72–2.02, *P-*value = 0.456; Δ32: OR = 1.33, CI = 0.41–4.46, *P*-value = 0.596).

### 3.3 CCR5Δ32 mutation and risk of breast cancer

#### 3.3.1 Amplification results

**Figure 2:**
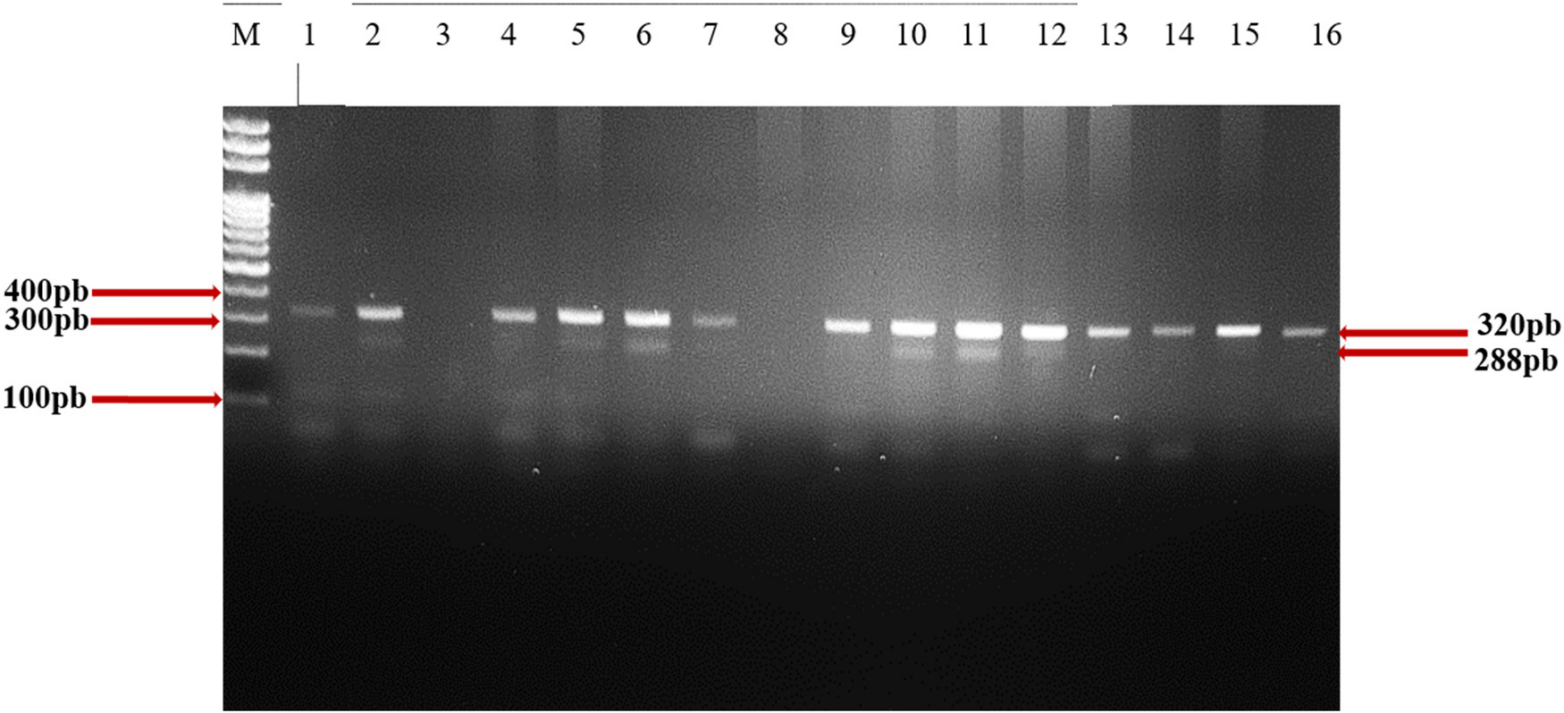
Electrophoretic profile of CCR5Δ32 mutation after migration. Numbers 1 to 16 represent samples. Samples 2, 5, 6, 10, 11, and 12: heterozygous 288 bp and 320 bp, and samples 1, 4, 7, 9, 13, 14, 15, 16: homozygous wild type 320 bp. Samples 3 and 8: no migration.

After conventional PCR amplification, 320 bp bands were obtained. This is the wild-type (WT) allele. Heterozygotes (WT/Δ32) had 320 and 288 bp and mutated homozygotes (Δ32/Δ32) had 288 bp (Figure 2).

#### 3.3.2 Genotypic and allelic frequencies

Genotypic frequencies were consistent with HWE between patients (χ² = 1.125, *P*-value = 0.28) and controls (χ² = 1.16, *P*-value = 0.288). The Δ32 mutant allele of this polymorphism was more prevalent in 6.25% of the patients than in controls (4.9%) (*P*-value = 0.596) (Table 4). In the general population, the genotypic frequencies of homozygotes (WT/WT) and heterozygotes (WT/Δ32) were 87.5% and 13.5%, respectively, but no mutated homozygotes (Δ32/Δ32) were found. In particular, these same genotypes (WT/WT and WT/Δ32) were represented in patients (87.5%, 13.5%) and controls (89.29%, 10.71%), respectively. No Δ32/Δ32 homozygotes were observed. Also, no significant association was found between variants of this polymorphism and breast cancer (OR = 1.33, 95% CI = 0.41–4.460, *P-*value = 0.596).

**Table 4:** Association between socio-characteristic and genotypes of the two polymorphisms

Genotypes	*TP63 (rs17506395)*		OR (95% CI)	*P*-value
	Cases N (%)	Controls N (%)		
**Pre-menopausal**				
TT	18 (25.71)	25 (37.88)	—	Reference
TG	52 (74.29)	41 (62.12)	1.76 (0.80–3.92)	0.127
GG	0 (0.00)	0 (0.0)	NA	NA
***CCR5*** **Δ32**				
WT/WT	61 (87.14)	59 (81.94)	—	Reference
WT/Δ32	9 (12.86)	13 (18.06)	0.67 (0.23–1.84)	0.392
Δ32/Δ32	0 (0.00)	0 (0.0)	NA	NA
**Post-menopausal**				
TT	2 (100)	1 (16.67)	—	Reference
TG	0 (0.0)	5 (83.33)	NA	NA
GG	0 (0.0)	0 (0.0)	NA	NA
***CCR5*** **Δ32**				
WT/WT	2 (100)	6 (100)	—	Reference
WT/Δ32	0 (0.0)	0 (0.0)	NA	NA
Δ32/Δ32	0 (0.0)	0 (0.0)	NA	NA

Genotypes	Family history (%)		OR (95% CI)	*P*-value
	Yes (*N* = 11)	No (*N* = 61)		
***TP63*** **(rs17506395)**				
TT	4 (36.36)	16 (26.23)		Reference
TG	7 (63.64)	45 (73.77)	0.62 (0.16–2.41)	0.74
GG	0 (0.00)	0 (0.0)	NA	NA
***CCR5*** **Δ32**				
WT/WT	10 (90.90)	53 (86.88)		Référence
WT/Δ32	1 (9.10)	8 (13.12)	0.66 (0.07–5.89)	1
Δ32/Δ32	0 (0.0)	0 (0.0)	NA	NA

Genotypes	Age at diagnostic (ans)		OR (95% CI)	*P* value
	Before 40 years	After 40 years		
***TP63*** **(rs17506395)**				
TT	3 (18.75)	5 (23.81)	—	Reference
TG	13 (81.25)	16 (76.19)	1.35 (0.21–10.30)	0.71
GG	0 (0.00)	0 (0.0)	NA	NA
***CCR5*** **Δ32**				
WT/WT	12 (75)	20 (95.24)		Reference
WT/Δ32	4 (25)	1( 4.76)	6.66 (0.54–34.28)	0.074
Δ32/Δ32	0 (0.0)	0 (0.0)	NA	NA

OR: odds ratio; 95% CI: 95% confidence interval, NA: not applicable.

### 3.4 Multivariable logistic analysis: association between certain characteristics and SNP rs17506395 (T > G) in *TP63* and CCR5Δ32 mutation

No significant association was found between the different genotypes of the polymorphisms and menopausal status (OR = 1.76, 95% CI = 0.80–3.92, *P*-value = 0.127), family history (OR = 0.62, 95% CI = 0.16–2.41, *P*-value = 0.74), and age at diagnosis (OR = 1.35, 95% CI = 0.21–10.30, *P*-value = 0.71) (Table 4).

### 3.5 Combined genotypes of SNP rs17506395 (T > G) in *TP63* and CCR5Δ32 mutation and breast cancer risk

The combined effect of polymorphisms on breast cancer risk has been demonstrated with several combined genotypes. However, no significant association was found between the combined genotypes of the polymorphisms and breast cancer (Table 5).

**Table 5:** Analysis of combined genotypes

TP63	Gene CCR5											
	WT/WT				WT/Δ32				Δ32/Δ32			
	C	T	OR (95% CI)	P	C	T	OR (95% CI)	P	C	T	OR (95% CI)	*p*
	N	N			N	N			n	N		
TT	13	14	—	Réf	3	2	1.62 (0.16–21.94)	0.626	0	0	NA	NA
TG	39	31	1.35 (0.5–3.63)	0.5	5	2	2.69 (0.35–32.06)	0.272	0	0	NA	NA
GG	0	0	NA	NA	0	0	NA	NA	0	0	NA	NA

T: controls; C: cases; N: number; Ref: reference; OR: odds ratio; CI: confidence interval; NA: not applicable.

## 4 Discussion

### 4.1 Socio-demographic and clinical characteristics

Our study population ranged in age from 19 to 70 years, with an average of 41.08 ± 12.19 years. Patients had a mean age of 46.22 ± 11.24 years; this mean was close to that of Zouré et al. in 2018 in Burkina Faso (47.4 ± 1.11 years) [21], whereas it differs from those found in Cameroon (41.64 years ± 12.31) (Tiofack et al., 2020) and (49.9 years ± 18.1) in Burkina Faso [28]. The mean age at diagnosis of breast cancer was 40.39 ± 10.96 years in our study. This average is lower (48.27 years and 50.5 years) than that found by Aka et al. [29] in Ivory Coast and Egypt in 2012 [30]. This difference could be justified by the fact that Burkina Faso’s population is characterised by its youth: around 80% of the population is aged under 35, according to the latest census published in 2020 by the National Institute of Statistics and Demography [31].

No significant association was observed between family history and the development of breast cancer in this study. These results differ from those of Antoniou et al. [31] and Economopoulou et al. [32], who were able to establish a link between family history and breast cancer.

Our results showed a significant association between BMI and the occurrence of breast cancer (OR = 3.8, 95% CI: 1.13–14.38, *P-*value = 0.015). These results are consistent with those found in other studies in France [2,33]. The probable explanation could be an accumulation of intra-abdominal fat by these patients. This accumulation of fat leads to obesity, the causes of which are essentially linked to bad eating habits such as fast food, unlicensed alcohol consumption, and physical inactivity (sedentary lifestyle). This obesity is more prevalent in urban areas due to the proximity of these food products. In fact, women living in urban areas had somewhat stable economic situations and could therefore indulge in an excessively high-fat diet. This excessive weight gain, combined with a sedentary lifestyle, could lead to the onset of breast cancer.

In reality, the link between obesity and breast cancer is complex [34]. Obesity induces metabolic dysfunctions, notably altered cellular metabolism and signalling pathways. It corresponds to excessive weight gain, and this intra-abdominal fat gain can lead to the formation of circulating oestrogens through an enzyme called aromatase. The accumulation of these oestrogens over a period of time in the body is thought to transform breast cells into cancer cells [35]. Obesity is also thought to be associated with hyperinsulinism, an increase in high glucose levels leading to resistance to breast cancer treatment, which in turn favours the appearance of tumours [36].

Late menopause was not associated with a risk of breast cancer. This difference in our results can be explained by the fact that there are fewer menopausal women in our study population. This result differs from those found by Moroccan [37] and Algerian [38] researchers, who showed that late menopause was linked to an increased risk of developing breast cancer. The main cause was found to be prolonged exposure to ovarian hormones in their study populations. The longer a woman waits for menopause, the more she is exposed to oestrogens, which are likely to promote the development of breast cancer. It is also important to note the use of steroid hormones and carcinogenic products by these same populations. They can acquire them through hormone replacement therapy [39]. The use of growth promoters for more than 5 years [40] and the use of hormonal contraceptives over a long period of time [41].

### 4.2 SNP rs17506395 (*TP63*) and CCR5Δ32 polymorphisms and breast cancer risk factors

Analysis of the genotypic and allelic frequencies of the study population showed that the different genotypes and alleles in the sample were in HWE for the Δ32 mutation of the *CCR5* gene. This was observed in both cases and controls (*P*-value > 0.05) for the CCR5 polymorphism (*x* ^2^ = 1.125, *P*-value = 0.28 and *x* ^2^ = 1.16, *P*-value = 0.288).

However, for the *TP63* gene, the population was not in equilibrium in either cases or controls (*x* ^2^ = 23.27, *P*-value = 0.000001 and *x* ^2^ = 16.74, *P*-value = 0.000043). In fact, Hardy–Weinberg’s law stipulates that within a population, allelic and genotypic frequencies remain constant from one generation to the next [42]. The allelic and genotypic frequencies of the Δ32 mutation in the *CCR5* gene are therefore constant from one generation to the next in Burkina Faso. However, the rs17506395 polymorphism does not respect Hardy Weinberg’s law. In 2020, in Cameroon, a study was able to demonstrate that their study population was indeed in equilibrium with *P-*value = 1 [8]. This difference could be explained by the relatively small size of our study population and also by the heterogeneity of this polymorphism (rs17506395). No association was observed between the rs17506395 polymorphism of the *TP63* gene and the development of breast cancer in the study population (*P-*value > 0.05). Our results corroborate those found in Cameroon that tried to elucidate the involvement of this polymorphism in the occurrence of breast cancer but who did not find a link between this polymorphism and breast cancer regardless of the genotype considered, while another Cameroonian study found a link between the TP63 polymorphism and people aged under 40 (OR = 0.5, 95% CI = 0.26–0.94, *P-*value = 0.03) [8]. Our results differ from those found in a Pakistani and Asian population, respectively, which had established an association between the TP63 polymorphism and the risk of developing breast cancer. Indeed, according to Fatima et al. in 2019 [43], the WT/Δ32 genotype was associated with an increased risk of developing breast cancer in Pakistan and the associated T allele would increase tumourigenicity because the TT genotype is associated with tumour amplification, making it a potential biomarker for an unfavourable prognosis in patients under 40 years of age [12,44]. According to a study in the same Asia, the GG mutant genotype of the TP63 polymorphism conferred protection against breast cancer [45]. This genotype was not encountered in our study.

We found no association between TG genotype and the development of breast cancer (OR = 1.47, 95% CI = 0.69–3.17, *P*-value = 0.284). In China, on the other hand, we found an association between breast cancer and the TG genotype in younger people [12]. The discrepancy between these results and those of the present study could be explained by the fact that the majority of our patients were over 40 years of age. In our study, the frequency of the T allele was higher than that of the G allele, although this difference was not statistically significant. These results are similar to those obtained in 2018 [46].

### 4.3 Association between certain socio-characteristics and polymorphisms (rs17506395 (*TP63*) and CCR5Δ32)

Our results show that there was no association between the TG genotype of the TP63 polymorphism or the WT/Δ32 genotype of the *CCR5* gene and the risk of breast cancer in pre-menopausal women or those with a family history of breast cancer. In terms of pre-menopausal status, these two genotypes had *P*-value = 0.127 and *P*-value = 0.392, while family history had *P*-value = 0.372 and *P* = 0.515. These data differ from those of a study carried out in the Cameroonian population [8] who found an association between these polymorphisms of the *TP63* gene and the Δ32 mutation of the *CCR5* gene and the occurrence of breast cancer in women with a family history. Also, in 2014, a study conducted in China [12] showed that TT and TG polymorphisms were significantly associated with an increased risk of breast cancer in women under 40. The likely hypothesis is that these patients have been exposed for a long time to steroid hormones and carcinogens that act on breast cells. This exposure can initiate tumourigenesis by causing DNA damage, such as mutations; in fact, prolonged exposure to hormones induces endocrine disruption, leading to the dysfunction of the hormonal system. This imbalance results in the proliferation of cancer cells. Exposure to carcinogens also causes mutations, notably in the BRCA 1 and 2 genes, which can lead to breast cancer. With regard to the family history of breast cancer and age at diagnosis, no risk was found between these factors and the genotypes of the rs17506395 polymorphisms of the *TP63* gene and the CCR5Δ32 mutation. These results are similar to those of the studies carried out in Cameroon [8] and Iran [44]. The same authors reported that there was no association between the rs17506395 (*TP63*) and CCR5Δ32 (*CCR5* gene) polymorphisms and age at diagnosis on the one hand, and family history of breast cancer on the other hand. In our study, most of the people included had no family antecedents, which may explain the absence of any link between family history and the occurrence of breast cancer in our context [8].

### 4.4 Combined effects of rs17506395 (*TP63*) and CCR5Δ32 polymorphism genotypes and breast cancer risk

We believe that the association between the two polymorphisms would have a greater influence on the occurrence of breast cancer than the effect of a single polymorphism. However, analysis of our data showed no significant association between the combined genotypes of these two polymorphisms and the occurrence of breast cancer (OR = 1.35, 95% CI = 0.55–3.29, *P-*value = 0.41). In the literature, to our knowledge, no other study in the past has shown a probable link between the genotypes of the rs17506395 polymorphisms of the *TP63* gene and the Δ32 mutation of the *CCR5* gene and breast cancer.

### 4.5 Limitations of our study

The limitations of our study can be summed up essentially not only by the small size of our sample but also by a lack of socio-demographic and clinical information. It would therefore be more interesting to continue the study on a larger population in order to assess more objectively the real impact of these two polymorphisms on the occurrence of breast cancer in Burkina Faso.

## 5 Conclusion

This first study in Burkina Faso reported the presence of the [G] mutant allele of the *TP63* gene in both cases and controls. Like the CCR5 Δ32 mutation, the [Δ32] mutant allele of the *CCR5* gene was present in both cases and controls. Neither the (TG) genotype of the *TP63* gene nor the (WT/Δ32) genotype of the *CCR5* gene was associated with the development of breast cancer in our study population.

## References

[1] Sung H, Ferlay J, Siegel RL, Laversanne M, Soerjomataram I, Jemal A, et al. Global Cancer Statistics 2020: GLOBOCAN estimates of incidence and mortality worldwide for 36 cancers in 185 countries. CA Cancer J Clin. 2021;71:209–49. 10.3322/caac.21660. 33538338

[2] Key TJ, Verkasalo PK, Banks E. Epidemiol Breast Cancer. Lancet Oncol. 2001;Mar;2(3):133–40. 10.1016/S1470-2045(00)00254-0 11902563

[3] Rojas K, Stuckey A. Breast cancer epidemiology and risk factors. Clin Obstet Gynecol. 2016;59:651–72. 10.1097/GRF.0000000000000239. 27681694

[4] Walsh T, Casadei S, Coats KH, Swisher E, Stray SM, Higgins J, et al. Spectrum of mutations in BRCA1, BRCA2, CHEK2, and TP53 in families at high risk of breast cancer. JAMA. 2006;295:1379. 10.1001/jama.295.12.1379. 16551709

[5] Shiovitz S, Korde LA. Genetics of breast cancer: A topic in evolution. Ann Oncol. 2015;26:1291–9. 10.1093/annonc/mdv022. PMC4478970 25605744

[6] Walsh T, King M-C. Ten genes for inherited breast cancer. Cancer Cell. 2007;11:103–5. 10.1016/j.ccr.2007.01.010. 17292821

[7] Guleria K, Sharma S, Manjari M, Uppal MS, Singh NR, Sambyal V. p.R72P, PIN3 Ins16bp polymorphisms of TP53 and CCR5Δ32 in North Indian breast cancer patients. Asian Pac J Cancer Prev. 2012;13:3305–11. 10.7314/APJCP.2012.13.7.3305. 22994752

[8] Tiofack ATZ, Simo G, Ofon E, Dina-Bell E, Kamla CM, Ananga SN, et al. The TP63 gene polymorphism rs17506395 is associated with early breast cancer in Cameroon. Asian Pac J Cancer Prev. 2020;21:2199–208. 10.31557/APJCP.2020.21.8.2199. PMC7771916 32856845

[9] Barbareschi M, Pecciarini L, Cangi MG, Macrì E, Rizzo A, Viale G, et al. p63, a p53 homologue, is a selective nuclear marker of myoepithelial cells of the human breast. Am J Surg Pathol. 2001;25:1054–60. 10.1097/00000478-200108000-00010. 11474290

[10] Feng Z, Zhang C, Kang H, Sun Y, Wang H, Naqvi A, et al. Regulation of female reproduction by p53 and its family members. FASEB J. 2011;25:2245–55. 10.1096/fj.10-180166. PMC3114525 21402718

[11] Guan X, Zhang N, Yin Y, Kong B, Yang Q, Han Z, et al. Polymorphisms in the p63 and p73 genes are associated with ovarian cancer risk and clinicopathological variables. J Exp Clin Cancer Res. 2012;31:89. 10.1186/1756-9966-31-89. PMC3542002 23095717

[12] Zhang N, Huo Q, Wang X, Chen X, Long L, Guan X, et al. A genetic variant in p63 (rs17506395) is associated with breast cancer susceptibility and prognosis. Gene. 2014;535:170–6. 10.1016/j.gene.2013.11.038. 24316488

[13] Yang A, Kaghad M, Wang Y, Gillett E, Fleming MD, Dötsch V, et al. p63, a p53 homolog at 3q27–29, encodes multiple products with transactivating, death-inducing, and dominant-negative activities. Mol Cell. 1998;2:305–16. 10.1016/S1097-2765(00)80275-0. 9774969

[14] Kaimen-Maciel D, Vissoci Reiche E, Brum Souza D, Frota Comini E, Bobroff F, Morimoto H, et al. CCR5-Δ32 genetic polymorphism associated with benign clinical course and magnetic resonance imaging findings in Brazilian patients with multiple sclerosis. Int J Mol Med. 2007;20(3):337–44. 10.3892/ijmm.20.3.337. 17671738

[15] Zafiropoulos A. Significant involvement of CCR2-64I and CXCL12-3a in the development of sporadic breast cancer. J Med Genet. 2004;41:e59-9. 10.1136/jmg.2003.013649. PMC1735773 15121787

[16] Zheng B, Wiklund F, Gharizadeh B, Sadat M, Gambelunghe G, Hallmans G, et al. Genetic polymorphism of chemokine receptors CCR2 and CCR5 in Swedish cervical cancer patients. Anticancer Res. 2006;Sep-Oct;26(5B):3669–74. 17094383

[17] von Luettichau I, Segerer S, Wechselberger A, Notohamiprodjo M, Nathrath M, Kremer M, et al. A complex pattern of chemokine receptor expression is seen in osteosarcoma. BMC Cancer. 2008;8:23. 10.1186/1471-2407-8-23. PMC2257957 18215331

[18] Mañes S, Mira E, Colomer R, Montero S, Real LM, Gómez-Moutón C, et al. CCR5 expression influences the progression of human breast cancer in a p53-dependent manner. J Exp Med. 2003;198:1381–9. 10.1084/jem.20030580. PMC2194244 14597737

[19] Aoki MN, da silva do amaral Herrera AC, Amarante MK, do Val Carneiro JL, Fungaro MHP, Watanabe MAE. CCR5 and p53 codon 72 gene polymorphisms: Implications in breast cancer development. Int J Mol Med. 2009;23:429–35. 10.3892/ijmm_00000148. 19212663

[20] Weng C-J, Chien M-H, Lin C-W, Chung T-T, Zavras A-I, Tsai C-M, et al. Effect of CC chemokine ligand 5 and CC chemokine receptor 5 genes polymorphisms on the risk and clinicopathological development of oral cancer. Oral Oncol. 2010;46:767–72. 10.1016/j.oraloncology.2010.07.011. 20729133

[21] Zoure AA, Slaoui M, Bambara HA, Sawadogo AY, Compaoré TR, Ouédraogo NLM, et al. BRCA1 c.68_69delAG (exon2), c.181T > G (exon5), c.798_799delTT and 943ins10 (exon11) mutations in Burkina Faso. J Public Health Afr. 2018;Jul 6;9(1):663. 10.4081/jphia.2018.663. PMC6057717 30079159

[22] Dabre S, Zoure AA, Kiendrebeogo TI, Zongo N, Amegnona LJ, Sombié HK, et al. Involvement of p.R72P and PIN3 Ins16bp (TP53) polymorphisms and the I157T (CHEK2) mutation in breast cancer occurrence in Burkina Faso. Asian Pac J Cancer Biol. 2023;8(2):135–45. 10.21203/rs.3.rs-1693953/v1.

[23] Zouré AA, Amegnona LJ, Zongo N, Kiendrebeogo IT, Sorgho PA, Zongo FI, et al. Carriage of HLA-DRB1*11 and 1*12 alleles and risk factors in patients with breast cancer in Burkina Faso. Open Life Sci. 2021;16:1101–10. 10.1515/biol-2021-0113. PMC8511965 34712820

[24] Kiendrebeogo IT, Zoure AA, Zongo FI, Ouedraogo SY, Sawadogo AY, Amegnona J, et al. Screening of BRCA1 (c.5177_5180delGAAA rs80357867 and c.4986 + 6T > C rs80358086) and the BRCA2 (c.6445_6446delAT rs80359592) genes for breast cancer prevention in Burkina Faso. Ethiop J Health Sci. 2022;32:699–708. 10.4314/ejhs.v32i4.5. PMC9341024 35950060

[25] Kadanga E, Zouré AA, Zohoncon TM, Traoré L, Ky BD, Yonli AT, et al. Carriage of mutations R462Q (rs 486907) and D541E (rs 627928) of the RNASEL gene and risk factors in patients with prostate cancer in Burkina Faso. BMC Med Genomics. 2022;15:123. 10.1186/s12920-022-01279-9. PMC9161613 35655265

[26] Traoré ADD, Ky BD, Traoré L, Zohoncon TM, Zouré AA, Yonli AT, et al. Carriage of Ser217Leu and Ala541Thr Variants of ELAC2 Gene and Risk Factors in Patients with Prostate Cancer in Burkina Faso. Prostate Cancer. 2022;2022:1–9. 10.1155/2022/3610089. PMC9833931 36643816

[27] Adico MDW, Zouré AA, Sombié HK, Kiendrebeogo TI, Dabré S, Amegnona LJ, et al. Involvement of ERCC1 ( rs3212986) and ERCC2 ( rs1799793, rs13181) polymorphisms of DNA repair genes in breast cancer occurrence in Burkina Faso. Molec Gen Gen Med. 2023;Apr;11(4):e2134. 10.1002/mgg3.2134. PMC10094077 36594475

[28] Zongo N, Ouédraogo A, Ouédraogo S, Sanon-Lompo S, Ido FA, Djiguemdé A, et al. Incidences et evolution des frequences des cancers au burkina faso de 1988 a 2018 titre court: epidémiologie des cancers au burkina faso. 2021;25:15.

[29] Aka E, Horo A, Koffi A, Fanny M, Didi-Kouko C, Nda G, et al. Expérience africaine monocentrique de la prise en charge personnalisée des cancers du sein à Abidjan: défis et perspectives. Gynécologie Obstétrique Fertilité Sénologie. 2021;49:684–90. 10.1016/j.gofs.2021.03.001. 33677121

[30] Hussien YM, Gharib AF, Awad HA, Karam RA, Elsawy WH. Impact of DNA repair genes polymorphism (XPD and XRCC1) on the risk of breast cancer in Egyptian female patients. Mol Biol Rep. 2012;39:1895–901. 10.1007/s11033-011-0935-7. 21643959

[31] Antoniou A, Pharoah PDP, Narod S, Risch HA, Eyfjord JE, Hopper JL, et al. Average risks of breast and ovarian cancer associated with BRCA1 or BRCA2 mutations detected in case series unselected for family history: A combined analysis of 22 studies. Am J Hum Genet. 2003;72:1117–30. 10.1086/375033. PMC1180265 12677558

[32] Economopoulou P, Dimitriadis G, Psyrri A. Beyond BRCA: New hereditary breast cancer susceptibility genes. Cancer Treat Rev. 2015;41:1–8. 10.1016/j.ctrv.2014.10.008. 25467110

[33] Wenten M. Associations of weight, weight change, and body mass with breast cancer risk in hispanic and non-hispanic white women. Ann Epidemiol. 2002;12:435–44. 10.1016/S1047-2797(01)00293-9. 12160603

[34] Neuhouser Marian L, Aragaki AK, Prentice RL, Manson JE, Chlebowski R, Carty CL, et al. Overweight, obesity and postmenopausal invasive breast cancer risk. JAMA Oncol. 2015;1:611–21. 10.1001/jamaoncol.2015.1546. PMC5070941 26182172

[35] Argolo DF, Hudis CA, Iyengar NM. The impact of obesity on breast cancer. Curr Oncol Rep. 2018;20:47. 10.1007/s11912-018-0688-8. 29644507

[36] LeVee A, Mortimer J. The challenges of treating patients with breast cancer and obesity. Cancers (Basel). 2023;15:2526. 10.3390/cancers15092526. PMC10177120 37173991

[37] Bouaalloucha S, Asmouki H, Soummani A. Le profil épidémiologique et clinique du cancer du sein chez la femme au CHU Mohammed VI de Marrakech. 2012.

[38] Henaoui L, Meguenni K. Facteurs de risque du cancer du sein - Étude cas-témoins Wilaya de Tlemcen. Revue d'Épidémiologie et de Santé Publique. 2020;68(3):S139

[39] Collaborative Group on Hormonal Factors in Breast Cancer. Breast cancer and hormone replacement therapy: Collaborative reanalysis of data from 51 epidemiological studies of 52 705 women with breast cancer and 108 411 women without breast cancer. Lancet. 1997;350:1047–59. 10.1016/S0140-6736(97)08233-0. 10213546

[40] Chaput G, Sumar N. Thérapies endocriniennes contre les cancers du sein et de la prostate: Éléments essentiels en première ligne. Can Fam Phys. 2022;68:e120–6. 10.46747/cfp.6804e120. PMC9007135 35418402

[41] Mørch LS, Skovlund CW, Hannaford PC, Iversen L, Fielding S, Lidegaard Ø. Contemporary Hormonal Contraception and the Risk of Breast Cancer. N Engl J Med. 2017;377:2228–39. 10.1056/NEJMoa1700732. 29211679

[42] Hartl DL, Clark AG. Principles of population genetics. 4th ed. Sunderland, MA: Oxford University Press; 2007.

[43] Fatima F, Saleem S, Hameed A, Haider G, Ali Zaidi SA, Kanwal M, et al. Association analysis and allelic distribution of deletion in CC chemokine receptor 5 gene (CCR5Δ32) among breast cancer patients of Pakistan. Mol Biol Rep. 2019;46:2387–94. 10.1007/s11033-019-04699-6. 30848448

[44] Tajbakhsh A, Farjami Z, Nesaei-Bajestani A, Afzaljavan F, Rivandi M, Moezzi A, et al. Evaluating the association between CCR5delta32 polymorphism (rs333) and the risk of breast cancer in a cohort of Iranian population. Iran J Public Health. 2021;50:583–91. 10.18502/ijph.v50i3.5604. PMC8214612 34178806

[45] Lee K-M, Choi J-Y, Kang C, Kang CP, Park SK, Cho H, et al. Genetic Polymorphisms of Selected DNA repair genes, estrogen and progesterone receptor status, and breast cancer risk. Clin Cancer Res. 2005;11:4620–6. 10.1158/1078-0432.CCR-04-2534. 15958648

[46] Hunt SE, McLaren W, Gil L, Thormann A, Schuilenburg H, Sheppard D, et al. Ensembl variation resources. Database (Oxford). 2018;2018:12. 10.1093/database/bay119 PMC6310513 30576484

